# Safety Decision-Making for Autonomous Vehicles Integrating Passenger Physiological States by fNIRS

**DOI:** 10.34133/cbsystems.0205

**Published:** 2025-05-13

**Authors:** Xiaofei Zhang, Haoyi Zheng, Jun Li, Zongsheng Xie, Huamu Sun, Hong Wang

**Affiliations:** ^1^ School of Vehicle and Mobility, Tsinghua University, Beijing 100084, China.; ^2^ Independent Researcher.

## Abstract

In recent years, several serious traffic accidents have exposed the severity of safety issues in autonomous driving technology. Traditional decision-making methods are unable to address potential risky behaviors caused by the functional insufficiencies or machine performance limitations, and human intervention is still needed. This study proposes an intelligent safety decision-making algorithm with passengers’ risk assessment by analyzing passenger physiological states online using functional near-infrared spectroscopy (fNIRS). This algorithm is developed based on twin-delayed deep deterministic policy gradient (TD3), and it can overcome the functional insufficiencies of traditional TD3 and guide TD3 using passengers’ risk assessment by analyzing passenger physiological states online while confronting risky scenarios. Three experiments have been conducted in autonomous emergency braking, front vehicle cutting-in, and pedestrian crossing scenarios. The results show that the proposed algorithm demonstrates faster convergence and superior safety and comfort performance compared with traditional TD3. This study highlights the applicability of fNIRS technology in enhancing the safety and comfort of autonomous vehicles in the future.

## Introduction

In recent years, there has been an increasing demand for enhancing the safety and reliability of autonomous vehicles. As of 2024 March 8, the California Department of Motor Vehicles had received 693 reports of autonomous vehicle accidents [1]. The insufficient functionality in terms of generalization, logical completeness, and rule coverage of autonomous driving algorithms are the critical elements of the safety of the intended functionality (SOTIF), which aims to ensure safety by considering limitations in performance and potential user misuse [2]. Traditional decision-making methods are unable to overcome potential risky behaviors caused by the intended functionality or performance limitations of machines. Since the machine can only provide limited sensory information for decision-making, decoding human cognitive information might be an alternative approach.

Functional near-infrared spectroscopy (fNIRS), a noninvasive real-time monitoring technique for measuring brain activity, is regarded as a unique source of cognitive information about driving [3]. Researchers have introduced this technology in autonomous driving for fatigue detection [4,5], trust evaluation [6], motion sickness studies [7], cognitive load assessments [8,9], cognitive engagement assessments [10], and studies on interactions with traffic [11]. Most of these studies were aimed at human drivers, and in the future, human drivers will take on the role of passengers for highly automated driving vehicles; thus, the risk perception of humans in high-level autonomous driving scenarios is essential. Perello-March et al. [12] conducted experiments with a driver-in-the-loop 3xD driving simulator to detect the brain hemodynamic responses of humans without engaging in driving. Their experimental results indicated that prefrontal cortical hemoglobin oxygenation levels substantially increased, following self-reported perceived risk and traffic complexity, particularly during hazardous scenario. In addition, Zhang et al. [13] established an fNIRS dataset for driving risk cognition of passengers in highly automated driving scenarios, reporting that the mental activities of passengers in the Brodmann area 10 are highly activated by driving risk [14] and high risk may result in the passengers’ mental activity on prefrontal cortex change [15].

Researchers have also been attempting to utilize brain signals to help machines understand human cognitive information for decision-making and control for various purposes, such as predicting limb movements [16,17] and recognizing emotions [18]. Specifically, in the realm of mobility, Teng et al. [19] studied the braking and steering intentions of humans using electroencephalogram (EEG) and distinguished the braking intentions of drivers at 420 ms after an emergency situation occurs, with an accuracy of 94%. Additionally, by combining steering intentions with decision algorithms based on rules and models, a model-based predictive shared control for vehicles was achieved [20]. Besides, Wang et al. [21] also designed a multitask-oriented brain-controlled intelligent vehicle system for the first time by integrating a novel neural decoding method of driver-secondary-task intention. Shin et al. [22] established a human–machine interaction system and an enhanced artificial intelligence decision algorithm based on error-related potentials, and this system could correct machine decisions using these potentials. Fu et al. [23] studied the EEG signals in highly automated driving scenarios and found that human braking intentions may be detected using EEG signals at 454 ± 234 ms before humans take a braking action; additionally, they concluded that there is an increase in the low-frequency activity of passengers’ brain EEG data in the cutting-in scenario [24]. The abovementioned brain–computer interface systems are all based on EEG. For fNIRS, Zhu et al. [25,26] proposed a method to detect drivers’ braking intentions, paving the way for future cognitive models for decision-making in autonomous driving. Previous studies based on fNIRS focus on signal analysis, especially the diver braking intention. In this paper, we make full use of the easy-to-wear characteristics and robustness to movement artifact advantages of fNIRS, and propose an intelligent safety decision-making algorithm with passengers’ risk assessment for autonomous vehicles based on our previous research findings, and the fusion of subjective perception of risk and intelligent algorithm TD3 was explored for the first time to the best of our knowledge. Delay characteristics of fNIRS make it difficult to avoid accidents by distinguishing diver braking intention using fNIRS. This study focuses on the identification of subjective risk, avoiding some aggressive explorations of TD3 and accelerating its learning speed. This brain–computer interface system provides the possibility of enhancing the safety and comfort of autonomous vehicles in the future.

This paper is structured as follows: The related knowledge of the proposed algorithm is introduced in Materials and Methods. In Experiments and Experimental Results, the detailed experimental procedures and the results of experiments are shown, respectively. Finally, we discuss our study’s findings in Discussion.

## Materials and Methods

**Fig. 1.**
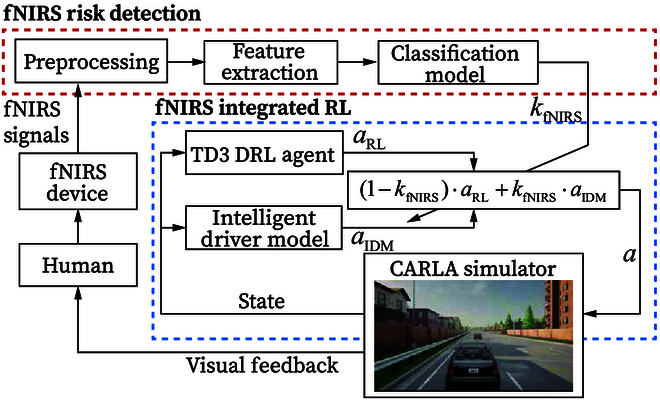
The overall diagram of this study.

The overall diagram is shown in Fig. 1. It contains 2 main parts: fNIRS risk detection and fNIRS integrated reinforcement learning (RL). The first part covers real-time preprocessing, feature extraction, and classification models. The second part focuses on the proposed human-guided deep reinforcement learning (DRL) scheme, and it contains a TD3 agent with some modifications, an intelligent driver model (IDM), and a human-guided DRL switching mechanism. This switching mechanism may accelerate the learning speed of TD3 based on passengers’ risk assessment using fNIRS.

### fNIRS risk detection

In this study, an online risk detection method is proposed, which aims to decode human perception of risk from the real-time collected fNIRS signals. First, 3 preprocessing steps need to be carried out, including baseline correction, resampling, and real-time filtering. Second, we extract features for the classification model via wavelet decomposition. Finally, a machine learning model is implemented based on a voting classifier to perform risk detection.

#### Preprocessing steps

The fNIRS signals collected in real time are subject to multiple sources of interference, including instrumental noises (such as external light in the surrounding environment), experimental noises (such as changes in the contact between the scalp and optodes), and, more importantly, physiological noises (including cardiac activity and blood pressure changes) [27]. To address this problem, we used a preprocessing method with 3 steps: baseline correction, resampling, and filtering.

First, 20 s of resting state data was collected before the onset of the stimulus, which was later used for baseline correction so that changes in hemoglobin concentration could be attained with our continuous-wave fNIRS device. Second, we resampled the signal with cubic spline interpolation at the sampling rate of 50 Hz to ensure that the signal was not affected by the unstable execution time. Finally, a double exponential moving average (DEMA) technique was used to exclude interference. In this study, these 3 steps were conducted on the concentration changes of deoxyhemoglobin (HHb) and oxyhemoglobin (O2Hb) separately.

The DEMA method is an improved version of the exponential moving average (EMA) method, which can be used for real-time filtering of fNIRS signals [28], and the DEMA method has a shorter lag before recursively updating the parameters to fit the current observations. The DEMA method is given as:

$$\begin{array}{cc} & {\text{DEMA}}_{\lambda }\left(n\right)=2y_{1}\left[n\right]-y_{2}\left[n\right]\\ & \begin{array}{cc} y_{1}\left[n\right]=\left(1-\lambda \right)\cdot y_{1}\left[n-1\right]+\lambda \cdot x\left[n\right] & \\ y_{2}\left[n\right]=\left(1-\lambda \right)\cdot y_{2}\left[n-1\right]+\lambda \cdot y_{1}\left[n\right] & \end{array} \end{array}$$ (1)

where ${\text{DEMA}}_{\lambda }\left[n\right]$ denotes the filtered signal and $x\left[n\right]$ denotes the input signal. Parameter λ is adjusted to achieve the desired cutoff frequency for this infinite impulse response (IIR) low-pass filter.

To achieve a bandpass filtering effect, we used it in a moving average convergence divergence (MACD) manner [29]. This was achieved by calculating the difference between 2 DEMA-filtered signals. The final output $y\left[n\right]$ is given as follows:

$$y\left[n\right]={\text{DEMA}}_{\lambda_{1}}\left[n\right]-{\text{DEMA}}_{\lambda_{2}}\left[n\right]$$ (2)

We tuned the parameters $\lambda_{1}$ and $\lambda_{2}$ as $\lambda_{1}=0.001$ and $\lambda_{2}=0.055$. The signal was further smoothed with a simple moving average technique, with the length of the averaging window set to 100 points. The cutoff frequencies were therefore approximated in MATLAB as 0.01 to 0.90 Hz. These cutoff frequency parameters were consistent with the most widely used band suggested by Klein and Kranczioch [30].

#### Feature extraction

In the filtering process, a longer signal series could result in better effects (because therefore the filter could make use of more past data points). So in preprocessing, we saved 5 s of data for DEMA filtering. Then, for feature extraction, we selected the latest preprocessed 4 s data. In this study, we utilized ΔCOE as an indicator of brain activity, defined as the difference between ΔHbR (HHb concentration change) and ΔHbO (O2Hb concentration change) divided by $\sqrt{2}$:

$$\Delta \mathrm{COE}=\frac{\Delta \mathrm{HbR}-\Delta \mathrm{HbO}}{\sqrt{2}}$$ (3)

First, these 4 s of ΔCOE data were subjected to Daubechies 4 (DB4) wavelet transform multilayer decomposition, followed by feature extraction and selection. In DB4 wavelet multi-layer decomposition, the signal is recursively convolved with a set of scaling coefficients $g\left[k\right]$ and wavelet coefficients $h\left[k\right]$, and then downsampled to ^1^/_2_ of its original frequency. In this study, the windowed signal from each channel (8 channels in total) could be decomposed into 4 levels, resulting in 4 sets of detail coefficients and 1 set of approximation coefficients. The decomposition at level a is calculated as:

$$\begin{array}{cc} x_{a,L}\left[n\right] & =\sum_{k}x_{a-1,L}\left[k\right]\cdot g\left[2n-k\right]\\ x_{a,H}\left[n\right] & =\sum_{k}x_{a-1,L}\left[k\right]\cdot h\left[2n-k\right] \end{array}$$ (4)

where k is a summation index, and $x_{a,L}\left[n\right]$ and $x_{a,H}\left[n\right]$ are the approximation coefficients and detail coefficients at level a decomposition, respectively. Note that $x_{a,L}\left[n\right]$ is the original signal if a=0.

Second, the wavelet decomposition results were then used for selecting features, and each set of coefficients was used for calculating 12 features, including: (1) the Shannon entropy [see Eq. (6)] [31]; (2 to 6) 5%,25%,75%,and95% percentiles and the median; (7) mean value; (8 and 9) standard deviation and variance; (10) root mean square; and (11 and 12) times of zero-crossing and mean-crossing. So for each channel, 60 features were extracted.

Finally, the top 4 features with the highest information gain were selected as the features for each channel. Therefore, we obtained 32 features (8 channels) for 4 s of data. Taking into account individual differences and scenario variations, the feature selection process for training the classification model in each scenario in this study was conducted independently. Information gain measures the extent of a particular feature that reduces the uncertainty of its categories in a dataset [31]. The information gain I(X;Y) of the random variable Y (classes) for another random variable X (features) is defined as follows:

$$I\;\left(X;Y\;\right)=H(Y)-H\;(Y|X)$$ (5)

which is the difference between the entropy $H\left(Y\right)$ and the conditional entropy H(Y|X). The entropy and the conditional entropy are defined as Eqs. (6) and (7), where $p\left(y_{i}\right)$ denotes the probability of $y_{i}$ over all possible values Y, and $p\left(y_{i}|x_{i}\right)$ denotes the prior probability of $y_{i}$.

$$H\left(Y\right)=-\sum_{y_{i}\in Y}\left(p\left(y_{i}\right)\cdot \text{log}\left(p\left(y_{i}\right)\right)\right)$$ (6)

$$H\left(Y|X\right)=-\sum_{x_{i}\in X}p\left(x_{i}\right)\sum_{y_{i}\in Y}\left(p\left(y_{i}|x_{i}\right)\cdot \text{log}\left(p\left(y_{i}|x_{i}\right)\right)\right)$$ (7)

#### Classification

In this study, machine learning classification algorithms were deployed for risk detection using fNIRS signals. During data collection, participants were instructed to press a button to indicate their subjective perception of risky or nonrisky states, which served as manual labels. The 32-dimensional feature vectors obtained during the feature extraction process were used as features, and the manually labeled data collected during the experiment were used as labels, transforming the risk detection task into a binary classification problem. However, it was observed in our research process that individual algorithms often suffer from issues such as overfitting on the training set and poor generalization performance on new data. To address these challenges, an ensemble learning approach, specifically a voting classifier, was employed to improve the classifier’s generalization performance.

The voting classifier designed in this study combined the predictions of the 6 base classifiers through soft voting. Adaptive boosting was employed for some of the base classifiers. The base classifiers are shown in Table 1.

**Table 1.** List of base classifiers

Number	Abbreviation	Algorithm
1	AB-DT	Adaptive Boosting Decision Tree Classifier
2	AB-GNB	Adaptive Boosting Gaussian Naive Bayes Classifier
3	AB-SVM	Adaptive Boosting Support Vector Machine Classifier
4	AB-LR	Adaptive Boosting Logistic Regression Classifier
5	RF	Random Forest Classifier
6	MLP	Multilayer Perceptron (Neural Network) Classifier

The soft voting strategy was employed to combine multiple weak classifiers into a strong classifier. The soft voting algorithm calculates the average probabilities predicted by each classifier and compares the average probabilities of different classes to determine the final classification result. In this study, this approach allowed us to obtain information regarding whether the current situation was risky or not based on the fNIRS signals.

### fNIRS integrated RL

**Fig. 2.**
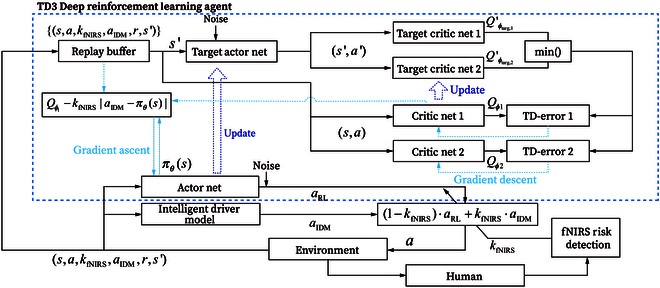
The fNIRS integrated RL diagram.

In this study, we designed a human-guided DRL switching mechanism. When the fNIRS signals indicated a risky state, the control would be switched to the IDM from the TD3 algorithm; at the same time, TD3 would learn from this decision-making. This could be beneficial in accelerating the learning speed of TD3 while maintaining exploratory performance. Moreover, with the guidance of fNIRS-induced protective measures, the RL agent was expected to learn action selection, such as deceleration, braking, or maintaining a safe distance from the preceding vehicle. The overall diagram of the system is shown in Fig. 2.

#### TD3 RL agent

TD3 RL method was introduced to improve the deep deterministic policy gradient (DDPG) algorithm by tackling the overestimation problem in value estimation [32]. DDPG algorithm is an actor-critic, model-free approach for RL to operate in continuous state and action space [33]. TD3 consists of 6 networks, with 2 networks forming a pair of actor networks, which include an actor network and a target actor network. The remaining 4 networks form 2 sets of critic networks. The actor network is responsible for learning the policy π that generates action a from state s. The critic network is used to calculate the *Q* value of taking action a in the corresponding state s, serving as a criterion to evaluate the quality of actions. According to [32], the update rule for the parameters of the critic network $\phi_{i}$ (i represents the index of the network, i=1,2) can be given as follows:

$$\phi_{i}\leftarrow \phi_{i}-\alpha \cdot \nabla_{\phi_{i}}\frac{1}{|B|}\sum_{(s,a,r,s^{\prime })\in B}\left(Q_{\phi_{i}}(s,\;a)-\left(r+\gamma \cdot \underset{i=1,2}{\text{min}}\left(Q_{\phi_{\text{targ},i}}^{\prime }\left(s^{\prime },\;a^{\prime }\left(s^{\prime }\right)\right)\right)\right)\right)$$ (8)

This is a gradient descent update process, with the learning rate α, where B is a batch of past observations sampled from the replay buffer, and ∣B∣ represents its size. $Q_{\phi_{i}}$ is the *Q* value generated from the critic network i, and $Q_{\phi_{\text{targ},i}}^{\prime }$ is generated from the target critic network, whose parameters are $\phi_{\text{targ},i}$. The actor network, on the other hand, is updated via a gradient ascent process with the learning rate β.

$$\theta \leftarrow \theta +\beta \cdot \nabla_{\theta }\frac{1}{|B|}Q_{\phi_{1}}\left(s,\;\pi_{\theta }\left(s\right)\right)$$ (9)

Then, the target network parameters $\phi_{\text{targ},1},\phi_{\text{targ},2}$, and $\theta_{\text{targ}}$ could be updated with the following rule (ρ is a constant):

$$\begin{array}{r} \phi_{\text{targ},i}\leftarrow \rho \cdot \phi_{\text{targ},i}+\left(1-\rho \right)\cdot \phi_{i}\\ \theta_{\text{targ}}\leftarrow \rho \cdot \theta_{\text{targ}}+\left(1-\rho \right)\cdot \theta \end{array}$$ (10)

In our study, the state was defined as an 8-dimensional vector, consisting of the ego vehicle velocity in the x and y directions, the ego vehicle x and y positions, the front vehicle velocities in the x and y directions, and the front vehicle x and y positions. The vehicle action was a control variable that mapped the ego vehicle’s acceleration to the range $\left[-1,1\right]$.

#### Intelligent driver model

IDM is a longitudinal traffic flow model that essentially calculates the ego vehicle’s acceleration based on the preceding vehicle’s speed and distance. In this study, when the fNIRS signal detected a risk signal, the IDM action was activated as a safe action instead of executing the RL action. The IDM action can be calculated as follows [34]:

$$\begin{array}{c} \begin{array}{r} \frac{dv}{dt}={\overset{\cdot }{v}}_{\max}\left[1-{\left(\frac{v}{v_{0}}\right)}^{\delta }-{\left(\frac{x^{*}\left(v,\;\Delta v\right)}{\Delta x}\right)}^{2}\right]\\ x^{*}(v,\;\Delta v)=x_{0}+\text{max}\left[0,\;\mathrm{vt}+\frac{v\Delta v}{2\sqrt{{\overset{\cdot }{v}}_{\max}\;\cdot \;{\overset{\cdot }{v}}_{\min}}}\right] \end{array} \end{array}$$ (11)

where v, Δx, and Δv denote ego vehicle’s velocity and the relative distance and speed between the ego vehicle and the front vehicle, respectively; $v_{0}$ and $x_{0}$ are the desired speed and the desired distance; ${\overset{\cdot }{v}}_{\max}$ and ${\overset{\cdot }{v}}_{\min}$ are the maximum acceleration and deceleration, respectively. δ is set as a constant 4.

#### Human-guided DRL switching mechanism

In this study, we designed a human-guided DRL switching mechanism, which is shown in Eq. (12)

$$a=\left(1-k_{\text{fNIRS}}\right)\cdot a_{\mathrm{RL}}+k_{\text{fNIRS}}\cdot a_{\mathrm{IDM}}$$ (12)

where $k_{\text{fNIRS}}$ is a boolean variable and has its value equal to 1 if the classification result “risky” has been detected; otherwise, the value is equal to 0; $a_{\mathrm{RL}}$ is the action generated by the TD3 agent. It can be seen from Eq. (12) that if a risk state occurs, the final action a taken is switched to IDM.

In order to accelerate the learning speed of TD3 and achieve human-guided DRL, it was necessary to introduce action imitation into the parameter update rule of the actor network. This involved performing gradient descent learning on the difference between the actions, which was used as an auxiliary behavior cloning loss suggested by Nair et al. [35]. Therefore, the adjustment to the parameter update rule [Eq. (9)] for the actor network is given as follows:

$$\theta \leftarrow \theta +\beta \cdot \nabla_{\theta }\frac{1}{|B|}\left[Q_{\phi_{1}}\left(s,\;\pi_{\theta }\left(s\right)\right)-k_{\text{fNIRS}}\cdot \left|a_{\mathrm{IDM}}-\pi_{\theta }\left(s\right)\right|\right]$$ (13)

where $\pi_{\theta }$ is the policy output by the current actor network.

### Experiments

#### Ethics statement

This study complied with the Declaration of Helsinki and later amendments of it. The content and procedures of this study were approved by the Institutional Review Board of Tsinghua University.

In this study, 10 participants (5 males and 5 females, aged 18 to 29) finished this experiment, and they needed to take part in 2 tasks: Collecting training data for the classification model and human-in-loop RL training. In the experiments, the participants were asked to watch the vehicle rendered in the CARLA simulator [36] in 3 driving scenarios. The participants’ information can be found in Table 2.

**Table 2.** Experiment participant information

No.	Sex	Age	Driving experience	Collected fNIRS data size (window slices, channels, data points)			Objective risk evaluation		
				Scenario no.			Scenario no.		
				1	2	3	1	2	3
S1	M	23	None	(1,800, 8, 200)	(964, 8, 200)	(795, 8, 200)	3	4	2
S2	M	19	None	(1,771, 8, 200)	(984, 8, 200)	(814, 8, 200)	3	4	3
S3	M	19	None	(1,766, 8, 200)	(983, 8, 200)	(802, 8, 200)	3	4	4
S4	M	21	None	(1,752, 8, 200)	(986, 8, 200)	(785, 8, 200)	4	5	3
S5	M	23	None	(1,762, 8, 200)	(998, 8, 200)	(808, 8, 200)	3	4	3
S6	F	18	None	(1,724, 8, 200)	(1,004, 8, 200)	(824, 8, 200)	3	4	2
S7	F	18	None	(1,775, 8, 200)	(965, 8, 200)	(802, 8, 200)	3	4	3
S8	F	19	None	(1,766, 8, 200)	(981, 8, 200)	(821, 8, 200)	4	3	3
S9	F	22	None	(1,765, 8, 200)	(985, 8, 200)	(821, 8, 200)	3	4	5
S10	F	29	None	(1,721, 8, 200)	(991, 8, 200)	(766, 8, 200)	4	3	4
Mean risk evaluation (1–5: “Very low”–“Very high”):							3.3	3.9	3.2

#### Experimental device

We used an OctaMon 2 × 4 channel (Artinis, the Netherlands) continuous wave (CW) NIRS device, consisting of 8 active channels (8 light-emitting diodes as transmitters Tx1 to Tx8, along with 2 photodiodes as detectors Rx1 and Rx2). The signals were acquired by the device and communicated via LSL (lab streaming layer) data streaming. The nominal wavelengths were $\lambda_{1|2}=760|850\;\mathrm{nm}$.

#### Experimental scenarios

**Fig. 3.**
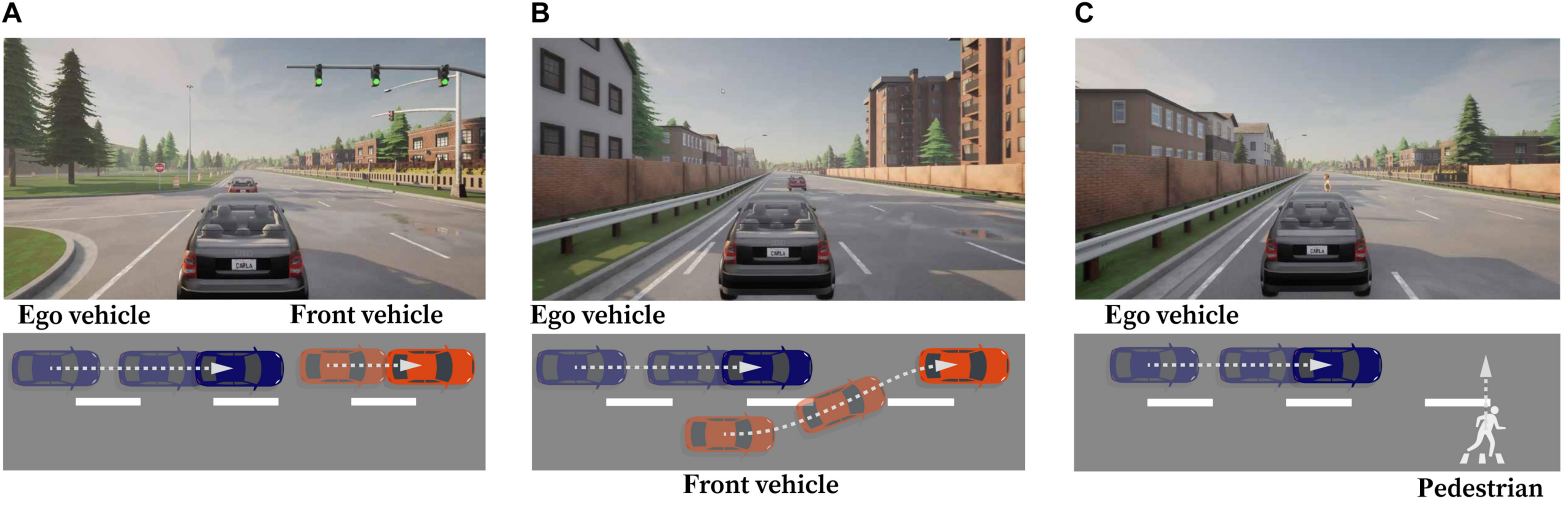
Three scenarios. (A) Scenario 1: AEB scenario. (B) Scenario 2: front car cutting-in scenario. (C) Scenario 3: pedestrian crossing scenario.

Three different scenarios were developed in this study, namely, an autonomous emergency braking (AEB) scenario (scenario 1), a front car cutting-in scenario (scenario 2), and a pedestrian crossing scenario (scenario 3), as shown in Fig. 3. Each scenario contained 10 s of relatively stable driving process and a risk situation with some randomness.

In scenario 1, the velocities of the ego vehicle and front vehicle were set to be 50 km/h at the beginning, and the initial distance between the 2 vehicles was set to be 50 m. After 10 s, the speed of the front vehicle decreased. The front vehicle would brake suddenly at a random relative distance ranging from 20 to 40 m. At this time, the ego vehicle needed to stop before colliding with the front car.

In scenario 2, the ego vehicle’s initial speed was set to 50 km/h and the front vehicle’s speed was set to 40 km/h. The initial longitudinal distance was 80 m. The front vehicle (which drove on a neighboring lane) started cutting in at a random time after 10 s. To avoid colliding, the ego vehicle needed to slow down in time.

In scenario 3, the ego vehicle’s speed was set to be 40 km/h, and a pedestrian would cross the road at a position 300 m ahead, at a random time after 10 s. The ego vehicle needed to decelerate in advance, avoiding getting too near to the pedestrian (within 1.5 m).

We also collected the participants’ objective risk evaluation of the 3 scenarios via 5-level Likert scale questionnaires, and risk levels 1 to 5 in our questionnaires indicated “Very Low”, “Low”, “Medium”, “High”, and “Very High”, respectively, as shown in Table 2. According to the participants’ feedback, scenario 2 had a higher level of risk than the other scenarios.

#### Experimental protocol

**Fig. 4.**
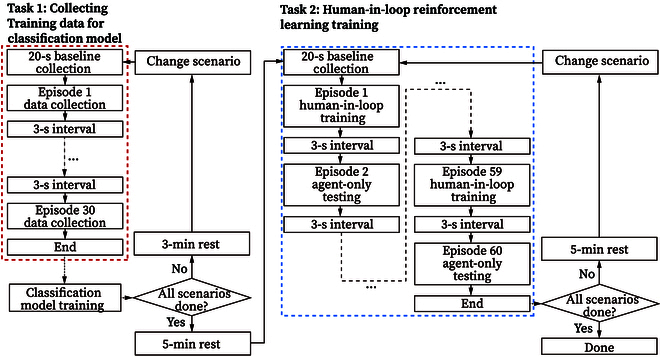
The experiment protocol.

The overall experimental protocol is shown in Fig. 4. In task 1, each volunteer participant was first shown a static scene in the simulator, and 20 s of resting baseline data was collected. Then, participants were asked to press the space button on the keyboard when they felt risky subjectively, and their subjective labels of risky situations were collected for later training. All 3 scenarios were shown to the participants, with 30 episodes for each scenario. After each episode, 3 s of frozen scene was shown to the participant as an interval. In each episode of data collection, numerous 5-second slices of fNIRS data were gathered from 8 channels of our device, saved as 2 arrays (one for HHb and the other for O2Hb) with the dimensions of 8×250. Simultaneously, the boolean value indicating whether the button was pushed was stored as the training label for objective risk evaluation. The total number of slices was not constant due to the variable duration of each episode. After 30 episodes of collection in one scenario, the participants were given 3 min of rest before switching to another scenario or 5 min of rest before shifting to task 2.

In task 2, baseline collection was first conducted in the same manner as in task 1. Then, the human-in-loop RL training began. In 2n−1,n∈N+ episodes, the participant’s fNIRS signal was used, and when the voting classifier identified risk via fNIRS signal, the control switched to IDM, guiding the agent to learn (noted as a “human-in-loop training episode”). This was followed by a testing episode, in which no fNIRS-induced guidance would override the agent’s behavior (noted as an “agent-only testing episode”) so that the effect of our proposed intelligent safety decision-making algorithm’s learning process could be verified. The duration of each episode was not constant but was always longer than 10 s because the first 10 s were set to be stable driving so that the participant could experience a gradual process of becoming nervous. Three seconds of intervals were added between episodes. After 30 human-in-loop episodes and 30 agent-only testing episodes, participants were given 5 min to rest before we switched to another scenario and restarted the process. The overall length of the experiment (including tasks 1 and 2) was approximately 1 hour 40 min for each participant.

## Results

### Task 1: Collecting training data for classification model

**Fig. 5.**
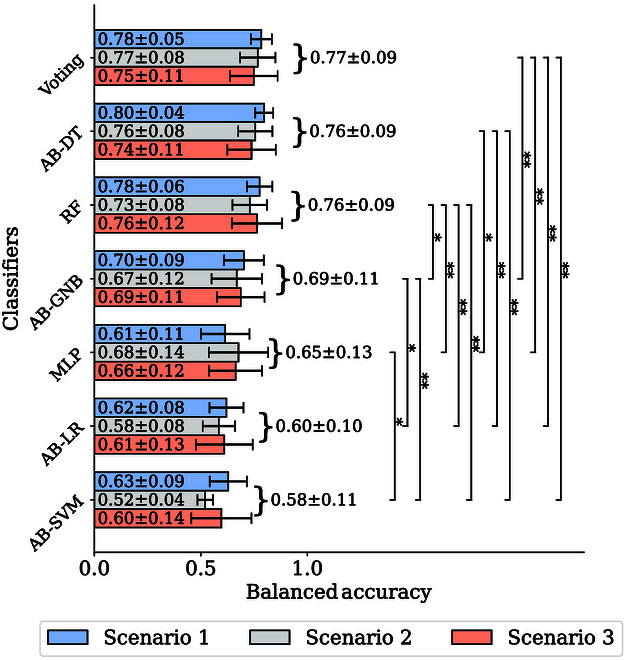
The overall BA of different classifiers in 3 scenarios. Asterisks denote significant effects at *P < 0.05, **P < 0.01, ***P < 0.001.

Classifier models were trained and tested separately on different subject individuals. Moreover, considering the unbalanced distribution of risk/safety labels in the datasets, we used balanced accuracy (BA) as the accuracy metric. The results are shown in Fig. 5. BA is defined as follows:

$$\mathrm{BA}=\frac{1}{2}\left(\frac{\mathrm{TP}}{\mathrm{TP}+\mathrm{FN}}+\frac{\mathrm{TN}}{\mathrm{TN}+\mathrm{FP}}\right)$$ (14)

where TP, TN, FP, and FN stands for true positives, true negatives, false positives, and false negatives, respectively.

As shown in Fig. 5, the average BA across the 3 scenarios of the proposed voting classifier had reached 0.77 ± 0.09 and outperformed the other 6 base classifiers. Friedman test suggests that there are significant differences (*P* < 0.001) among the BA results of 7 classifier models. With post hoc Nimenyi test, the BA results of the voting classifier, AB-DT classifier, and RF classifiers showed significant statistical differences compared with MLP, AB-LR, and AB-SVM classifiers; besides, there is a statistical difference between the proposed voting approach and AB-GNB with higher significant level (*P* < 0.01).

### Task 2: Human-in-loop RL training

In this section, we evaluated the proposed algorithm by its learning curves and its performance in terms of safety and comfort.

#### Learning process

**Fig. 6.**
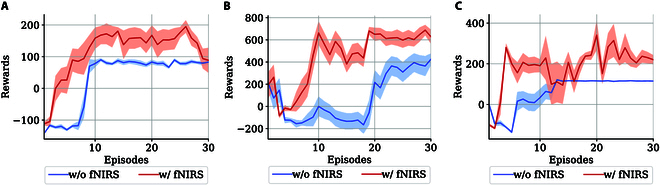
The reward curves in the 3 scenarios. The shaded area represents the standard deviation. (A) Scenario 1. (B) Scenario 2. (C) Scenario 3.

In Fig. 6, the cumulative reward curves are compared in 3 scenarios to demonstrate the effectiveness of introducing fNIRS risk detection in TD3, forming a human-in-loop RL scheme. The red line represents the rewards in testing episodes, demonstrating how the agent performed with the aid of the fNIRS-induced guidance. Compared with the blue cumulative reward curve of traditional TD3 under normal conditions, it is shown that the proposed intelligent safety decision-making algorithm with passengers’ risk assessment can accelerate the learning speed of RL agents by providing more predictive information about risky situations and demonstrating emergency avoidance actions to the agent.

#### Safety assessment

**Fig. 7.**
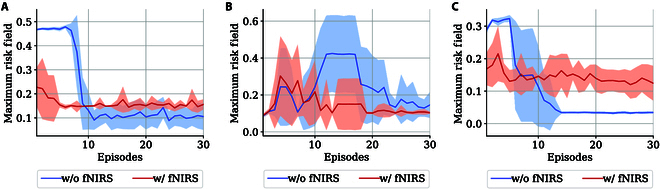
The maximum risk field value curve during training in 3 scenarios. The shaded area represents the standard deviation. (A) Scenario 1. (B) Scenario 2. (C) Scenario 3.

For the safety assessment of the learning process, we adopted the driving risk field to quantify the risk [37]. The kinetic energy field $E_{v}$ is calculated based on vehicle information, as shown below:

$$E_{v}=\frac{{\mathrm{GR}}_{2}M}{r^{k_{1}}}\frac{r^{k_{1}}}{|r^{k_{1}}|}e^{\left[k_{2}|v_{2}|\text{cos}\theta_{2}\right]}$$ (15)

where constants $k_{1}=1$, $k_{2}=0.05$, $R_{2}=1$, G=0.001, and M=1,705 are set according to [14]. r is the vector of relative position between the front vehicle and the ego vehicle. $v_{2}$ is the the other vehicle’s speed. $\theta_{2}$ represents the angle between r and $v_{2}$. The maximum risk value in each episode was plotted, as shown in Fig. 7. It is notable that the introduction of fNIRS risk detection effectively reduced the risk level in the exploration process.

For the safety assessment of the learning results, the minimum time-to-collision (*TTC*) index was also employed as the evaluation criterion. *TTC* is defined as the ratio of the distance between 2 vehicles $r\left(t\right)-l$ (l is the vehicle length) to the difference in their speeds $\overset{\cdot }{r}\left(t\right)$:

$$\mathrm{TTC}\left(t\right)=\frac{r\left(t\right)-l}{-\overset{\cdot }{r}\left(t\right)}$$ (16)

**Fig. 8.**
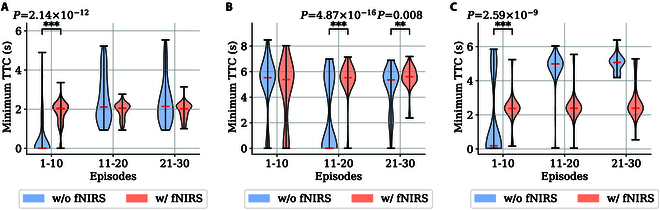
The distributions of minimum *TTC* observed in the 3 scenarios using violin plots, with one-sided Wilcoxon test *P* values shown on the top of each plot. (A to C) Minimum *TTC* observed in scenarios 1, 2, and 3, respectively. Asterisks denote significant effects at *P < 0.05, **P < 0.01, ***P < 0.001.

A smaller *TTC* value indicates a driving situation with more risk, and when *TTC* equals zero, it signifies that a collision has occurred. The initial 10, middle 10, and last 10 episodes were taken for comparison. The minimum *TTC* is a widely used indicator in the evaluation of safety driving [38,39], and its values in these episodes were plotted in violin plots, as shown in Fig. 8. Single-sided Wilcoxon tests were employed, with outliers removed using the robust *z*-score method with a threshold set at 3. It could be observed that in scenarios 1 and 3, there are a greater minimum *TTC* in the beginning for the groups with fNIRS with statistical significances. As for scenario 2, the minimum *TTC* with fNIRS risk detection is significantly higher in both episodes 11 to 20 and 21 to 30.

It can be also observed that the control group could have minimum *TTC* greater or equal to those with fNIRS-induced guidance (scenarios 1 and 3, episodes 11 to 20 and 21 to 30). However, by reviewing the reward curves in Fig. 6A and 6B, it can be noticed that the vehicle also received lower rewards in these episodes, indicating that the higher *TTC* achieved by the pure RL agent in these episodes is in fact a result of adopting an overly conservative strategy, representing a local optimum. For example, the RL agent often resorted to sudden braking from a significant distance away from the pedestrian.

#### Comfort assessment

For comfort assessment, the average jerk (including positive and negative) was used as the evaluation metric. The one-sided Wilcoxon test was used for statistical analysis. According to the literature [40,41], a large jerk can cause discomfort and is an indicator of aggressive driving, which can also threaten traffic safety. Jerk is defined as the derivative of acceleration $\overset{\cdot }{v}$:

$$\ddot{v}=\frac{d\overset{\cdot }{v}}{dt}$$ (17)

**Fig. 9.**
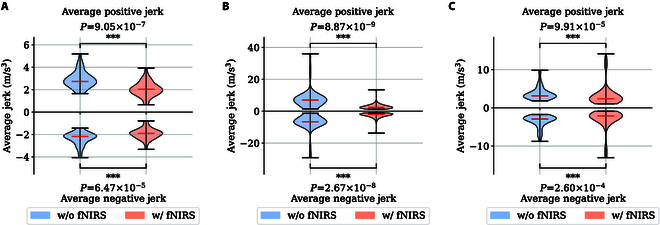
This figure shows the distribution of average positive/negative jerk observed in the 3 scenarios using violin plots. The vertical axis represents the average jerk, with the upper part of the horizontal axis indicating the average positive jerk and the lower part indicating the average negative jerk. (A to C) Scenarios 1 to 3, respectively. The one-sided Wilcoxon test *P* values are shown on each plot. Asterisks denote significant effects at *P < 0.05, **P < 0.01, ***P < 0.001

Based on Fig. 9, after the introduction of fNIRS, both negative jerk and positive jerk were reduced, indicating that the proposed intelligent safety decision-making algorithm performed better in terms of when and where to accelerate, decelerate, or brake, avoiding sudden maneuvers. According to the one-sided Wilcoxon signed rank test results, this effect was highly significant in those 3 scenarios.

## Discussion

Previous studies focus on signal analysis. Wang and colleagues [14,15] analyzed the relationship between risk and mental activity, and Zhu et al. [25] considered how to find diver braking intentions based on fNIRS. In this study, a voting classifier to identify fNIRS signal indicating risk was implemented, reaching an average BA of 0.77±0.09. We further integrated such physiological feedback into decision-making by using TD3 DRL with IDM guidance induced by fNIRS risk detection. The method demonstrated faster learning, safer exploration process, and better comfort performances.

In terms of risk detection with machine learning classifiers, we have noticed that both support vector machine and logistic regression base classifiers performed poorly in our tasks. We believe that this results from the limited capability of dealing with nonlinearity in the classification task, because both methods aim to classify data points by constructing a linear discriminant function that divides the feature space into distinct regions, and are constrained when dealing with nonlinearity due to computation complexity. Moreover, the real-time processing of fNIRS signals inherently introduces delays, leading to difficulties in promptly detecting risks in scenarios where risks rapidly increase, such as a sudden collision of the ego vehicle with the front vehicle at the maximum acceleration. Additionally, challenges may arise in applying our findings to more complex and realistic driving situations due to the relatively simplistic and short-lasting driving scenarios, as well as the narrow range of ages and homogeneous ethnicity among participants in our experiments.

As for the DRL decision-making process, due to the time constraints on wearing the fNIRS device for each participant, we limited the training process of the RL agent to 30 episodes. Unfortunately, this was insufficient for the agent to thoroughly explore the environment and discover the optimal policy. Particularly in scenario 3, the pure RL agent tended to adopt a suboptimal strategy of applying emergency brakes even when the pedestrian first appeared far ahead, resulting in unrealistic and overly conservative decision-making. Consequently, both the minimum TTC and risk field metrics displayed unsatisfactory outcomes. Enhancing the agent’s learning efficiency within a short training period remains a key focus for future research endeavors.

In future endeavors, we plan to deploy our algorithms in a broader spectrum of realistic and intricate driving scenarios. By integrating the risk detection outcomes derived from fNIRS signals with the sensory information of the vehicle, we aim to enhance the robustness and precision of driving risk estimation. Through comprehensive experiments, we intend to demonstrate the superior performance of the autonomous driving decision-making algorithm when augmented with fNIRS technology compared to conventional methods. Our ultimate goal is to harness this technology to enhance the safety standards of autonomous driving systems.

## Data Availability

The relational codes and data in this study have been uploaded on GitHub (https://github.com/haoyizheng2000/-Safety-Decision-Making-for-Autonomous-Vehicles-Integrating-Passenger-Physiological-States-by-fNIRS). Alternatively, please contact the corresponding author with any further queries regarding code or data availability.
